# Circulating Prostaglandin Biosynthesis in Colorectal Cancer and Potential Clinical Significance^☆^^☆☆^^★^

**DOI:** 10.1016/j.ebiom.2014.12.004

**Published:** 2014-12-09

**Authors:** Haitao Li, Kangdong Liu, Lisa A. Boardman, Yuzhou Zhao, Lei Wang, Yuqiao Sheng, Naomi Oi, Paul J. Limburg, Ann M. Bode, Zigang Dong

**Affiliations:** a The Hormel Institute, University of Minnesota, Austin, MN, USA; b The China –US (Henan) Hormel Cancer Institute, Zhengzhou, Henan, China; c The School of Basic Medical Sciences, Zhengzhou University, Zhengzhou, China; d The Affiliated Cancer Hospital, Zhengzhou University, Zhengzhou, China; e Division of Gastroenterology and Hepatology, Mayo Clinic, Rochester, MN, USA; f Department of Medicine, Mayo Clinic, Rochester, MN, USA

**Keywords:** CRC, colorectal cancer, FAP, familial adenomatous polyposis, PGs, prostaglandins, TXA_2_, thromboxane A_2_, mPGES1, microsomal prostaglandin E synthase-1, TBXAS1, thromboxane A_2_ synthase 1, TBXA2R, thromboxane A_2_ receptor, Colorectal cancer, Familial adenomatous polyposis, Thromboxane A_2_

## Abstract

**Background:**

Colorectal cancer (CRC) represents the third leading cause of cancer-related death in the United States. Lack of reliable biomarkers remains a critical issue for early detection of CRC. In this study, we investigated the potential predictive values of circulating prostaglandin (PG) biosynthesis in CRC risk.

**Methods:**

Profiles of circulating PG biosynthesis and platelet counts were determined in healthy subjects (n = 16), familial adenomatous polyposis (FAP) patients who were classified as regular aspirin users (n = 14) or nonusers (n = 24), and CRC patients with (n = 18) or without FAP history (n = 20). Immunohistochemistry staining was performed on biopsy samples.

**Results:**

Analysis of circulating PG biosynthesis unexpectedly revealed that CRC progression is accompanied by a pronounced elevation of circulating thromboxane A_2_ (TXA_2_) levels. When a circulating TXA_2_ level of 1000 pg/mL was selected as a practical cutoff point, 95% of CRC patients were successfully identified. Further study suggested that the TXA_2_ pathway is constitutively activated during colorectal tumorigenesis and required for anchorage-independent growth of colon cancer cells.

**Conclusions:**

This study established the importance of the TXA_2_ pathway in CRC pathophysiology, and laid the groundwork for introducing a TXA_2_-targeting strategy to CRC prevention, early detection and management.

## Highlights

• CRC progression is associated with higher circulating TXA_2_ levels, which merits investigation as a predictor of CRC risk.
• TXA_2_ pathway is constitutively activated during colorectal tumorigenesis.
• TXA_2_ pathway is required for anchorage-independent growth of colon cancer cells.
• This study lays the groundwork for introducing a TXA_2_-targeting strategy to CRC prevention, early detection and management.

## 1 Introduction

Colorectal cancer (CRC) represents the third leading cause of cancer-related death in the United States (Siegel et al., 2014a, Siegel et al., 2014b). Despite major improvements in preventive strategies and chemotherapeutic regimens, little change in CRC mortality has occurred over the past 50 years, which is at least partly due to late diagnosis (Srivastava et al., 2001). A lack of reliable biomarkers remains a critical issue for CRC early detection. Although colonoscopy screening and fecal occult blood testing have proven to be effective in the early detection of CRC, patient compliance is still low (deVos et al., 2009). Therefore, an urgent need exists to identify novel and convenient biomarkers for early detection of CRC.

Prostaglandins (PGs) and prostaglandin-endoperoxide synthases (PTGS, cyclooxygenases) have been implicated in various pathological processes such as inflammation, cardiovascular disease and cancer (Wang and Dubois, 2010). Although pivotal roles for colonic PGs and PTGS have been well-established in colorectal tumorigenesis (Castellone et al., 2005, Chulada et al., 2000, Oshima et al., 1996, Sonoshita et al., 2001), the profiles of circulating PG biosynthesis in CRC remain unclear. Herein, we investigated whether patients at high risk of developing CRC could be identified from their levels of circulating PGs.

## 2 Materials and Methods

### 2.1 Materials, Chemicals, and Reagents

Primary antibodies against human microsomal prostaglandin E synthase-1 (mPGES1), thromboxane A_2_ synthase 1 (TBXAS1), and thromboxane A_2_ receptor (TBXA2R) were obtained from Cayman Chemical Company (Ann Arbor, MI). All chemicals were purchased from Sigma-Aldrich (St Louis, MO) unless otherwise specified.

### 2.2 Cell Culture and Transfection

All cell lines used in this study were obtained from the American Type Culture Collection (ATCC, Manassas, VA) and maintained following ATCC instructions. Cells were cytogenetically tested and authenticated before being frozen. Each vial of frozen cells was thawed and maintained for a maximum of 20 passages. For lentiviral transfection, the jetPEI reagent (Qbiogene, Inc., Montreal, Quebec, Canada) was used, following the manufacturer's instructions. The 29-mer small hairpin RNA (*shRNA*) constructs against human *TBXA2R* and *TBXAS1* were obtained from Open Biosystems, Inc. (Huntsville, AL).

### 2.3 Anchorage-Independent Growth Assay

In each well of a 6-well plate, cells (8 × 10^3^) were suspended in Basal Medium Eagle (BME) medium (1 mL, with 10% FBS and 0.33% agar) and plated over a layer of solidified BME (3 mL, with 10% FBS and 0.5% agar). The cultures were incubated in a 37 °C, 5% CO_2_ incubator for 7 d and colonies in soft agar were counted under a microscope equipped with the Image-Pro Plus software program (Media Cybernetics, Bethesda, MD).

### 2.4 Western Blot Analysis

Protein samples (20 μg) were resolved by SDS-PAGE and transferred to Hybond C nitrocellulose membranes (Amersham Corporation, Arlington Heights, IL). After blocking, the membranes were probed with primary antibodies (1:1000) overnight at 4 °C. The targeted protein bands were visualized using an enhanced chemiluminescence reagent (Amersham Corporation) after hybridization with a secondary antibody conjugated with horseradish peroxidase.

### 2.5 Clinical Study

#### 2.5.1 Study Design

Volunteers were recruited by the Gastroenterology and Hepatology group at Mayo Clinic, Rochester, Minnesota or from the Gastroenterology group at The Affiliated Cancer Hospital, Zhengzhou University, Zhengzhou, China. All clinical studies using human subjects or human materials were approved by the Mayo Clinic review board or The affiliated Cancer Hospital, Zhengzhou University review board (#2014xjs28), respectively. Written, informed consent was required for entry of any patient into this study. Exclusion criteria included cigarette smoking, inflammatory bowel diseases, hypertension, a history of cardiovascular diseases, and pregnancy.

#### 2.5.2 Subjects

Individuals in the healthy control group (n = 16) were normal subjects who underwent colonoscopy screening. Familial adenomatous polyposis (FAP) patients who reported taking two or more standard (325 mg) aspirin tablets per week within the previous 12 months were classified as regular aspirin users (n = 14) and those reporting consumption of less aspirin were classified as aspirin nonusers (n = 24) (Chan et al., 2007). Individuals in the sporadic colorectal cancer group (n = 20) were patients who were diagnosed with CRC, but without a family history of CRC. The gender ratio in each group was approximately 1:1.

#### 2.5.3 Measurement of Plasma PGs

Briefly, blood was collected from a vein in the arm just inside the elbow using a 22 gauge needle. Before blood collection, the tourniquet was applied about three inches above the selected puncture site. Venous blood was drawn into a BD vacationer® PST™ plasma separation tube (#367964, BD Biosciences) containing lithium heparin. Blood samples were then centrifuged at 2000 ×*g* for 15 min and the resulting supernatant fraction was designated as plasma. The measurement of plasma PGs was performed using enzyme immunoassay kits from Cayman Chemical Company (Ann Arbor, MI) following the manufacturer's instructions. Considering the fact that PGD_2_, PGF_2α_, PGI_2_, and TXA_2_ are unstable in vivo, we determined their corresponding primary metabolites in plasma as follows: 11-beta-PGF_2α_, 13,14-dihydro-15-keto-PGF_2α_, 6-keto-PGF_1α_, and TXB_2_.

#### 2.5.4 Measurement of Urinary 11-Dehydro-Thromboxane B_2_

Urine samples were collected for determination of urinary 11-dehydro-thromboxane B_2_ (the major TXB_2_ metabolite) levels. Samples were collected from healthy (n = 8) or CRC patient (n = 24) volunteers recruited from the Gastroenterology group at The Affiliated Cancer Hospital, Zhengzhou University, Zhengzhou, China. All clinical studies using human subjects or human materials were approved by the The Affiliated Cancer Hospital, Zhengzhou University review board (#2014xjs28). Samples were collected between 8 p.m. and 8 a.m. and kept in − 80 °C. Determination of 11-dehydro-thromboxane B_2_ was performed by using an enzyme immunoassay kit (11-dehydro-thromboxane B_2_ EIA Kit, Cayman Chemical item number 519510) following the manufacturer's instructions. Urinary creatinine levels were detected by a Creatinine (urinary) Colorimetric Assay Kit (Cayman Chemical item number 500701) as an index of standardization for 11-dehydro-thromboxane B_2_ (Cayman Chemical item number 500701).

#### 2.5.5 Histology and Immunohistochemistry

Surgically resected human colon tissues at all clinical stages were fixed in 10% formalin overnight at room temperature. For histology, fixed tissues were embedded in paraffin, sectioned at 5 μm, and stained with hematoxylin and eosin (H&E) according to standard protocols. Immunohistochemistry staining for human cyclooxygenase-2 (COX-2, #12282, Cell Signaling Technology; dilution 1:200), mPGES1 (#160140, Cayman Chemical Company; dilution 1:50), TBXAS1 (#160715, Cayman Chemical Company; dilution 1:50), TBXA2R (#10004452, Cayman Chemical Company; dilution 1:50), or Ki-67 (RM-9106, Thermo Scientific, Fremont, CA; dilution 1:200) was performed using an ABC complex kit (PK-6100, Vector Laboratories, Burlingame, CA) following the manufacturer's instructions. Sections were counterstained with Harris's hematoxylin. For antibody-negative controls, the primary antibodies were substituted with normal rabbit serum. Immunohistochemistry staining intensity was quantified by calculating the integrated optical density (IOD, sum) of the area of interest using the Image Pro-Plus 7.0 software program.

### 2.6 Statistical Analysis

Statistical analysis was performed using the Prism 5.0 statistical software package. Pearson correlation was used to measure the strength of association between two variables. The Tukey's t-test was used to compare data between two groups. One-way ANOVA and the Bonferroni correction were used to compare data between three or more groups. Values are expressed as means ± S.D. and a *p* value of < 0.05 was considered statistically significant.

## 3 Results

### 3.1 Profiles of Circulating PG Biosynthesis in CRC

**Fig. 1:**
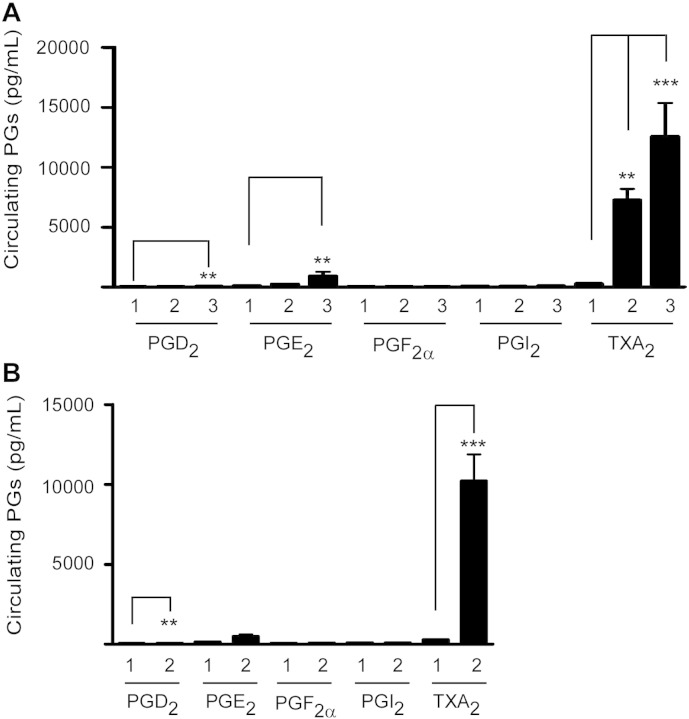
Circulating PG biosynthesis in CRC progression. (A) Circulating PG levels in healthy subjects or FAP patients. 1 = healthy subjects (n = 16); 2 = FAP patients with colonic adenomas (n = 24); and 3 = FAP patients with colonic adenocarcinomas (n = 18). Plasma samples were collected for measurement of circulating prostaglandin levels using an enzyme immunoassay kit (Cayman). Data are presented as means ± S.D. The asterisks indicate a significant (**, *p* < 0.01; ***, *p* < 0.001) difference compared to the group of healthy subjects. (B) Profiles of circulating PG biosynthesis in healthy subjects (1) or sporadic CRC (2) patients (n = 20). Data are presented as means ± S.D. The asterisks indicate a significant (**, *p* < 0.01; ***, *p* < 0.001) difference compared to the group of healthy subjects.

We first analyzed the profiles of circulating PG biosynthesis during CRC progression. The multistep nature of CRC (the so-called normal epithelial mucosa–adenoma–carcinoma sequence) has been well-established in FAP patients who universally develop CRC in the absence of colonic resection (Markowitz and Bertagnolli, 2009). Accordingly, we recruited FAP patients, and further sub-grouped them based upon pathological disease stage. Among the five major bioactive PGs examined, TXA_2_, but not PGE_2_, was the most abundant PG in plasma from FAP patients (Fig. 1A). Compared with healthy subjects, the levels of PGD_2_, PGE_2_ and TXA_2_ were significantly elevated in FAP patients, whereas PGF_2α_ and PGI_2_ levels did not change significantly. Intriguingly, circulating PGD_2_ and PGE_2_ were moderately elevated at the rather late stage (the adenoma–carcinoma sequence), whereas circulating TXA_2_ was dramatically elevated throughout the entire progression of CRC in FAP patients. For example, in FAP patients who had developed CRC, circulating TXA_2_ levels were strikingly increased to 44.3-fold of the normal level, but circulating PGE_2_ levels were only enhanced by 6.7-fold.

We next analyzed the profiles of circulating PG biosynthesis in sporadic CRC patients. Similar results were obtained (Fig. 1B). Of the five PGs measured, TXA_2_ was present at the highest concentration and only the levels of TXA_2_ were significantly elevated in sporadic CRC patients compared with healthy subjects. The circulating TXA_2_ levels in sporadic CRC patients were 35.9-fold higher than the normal level. These results indicate that, overall, CRC is accompanied by a pronounced elevation of the level of circulating TXA_2_.

### 3.2 Prognostic Value of Circulating TXA_2_ Levels in CRC

**Fig. 2:**
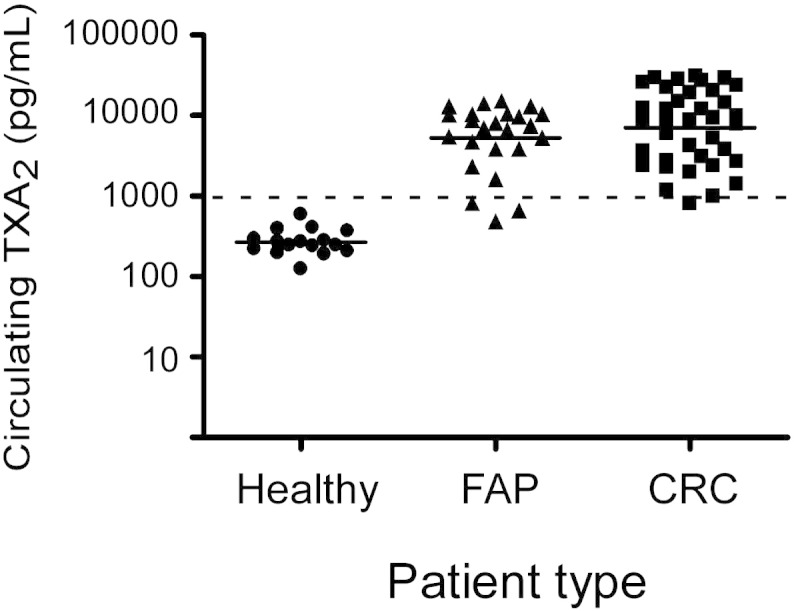
Prognostic value of circulating TXA_2_ levels in CRC. To confirm the prognostic value of circulating TXA_2_ levels in CRC, a test study was conducted in healthy subjects (n = 16), FAP patients (n = 24), and CRC patients with (n = 18) or without FAP history (n = 20). Based on a value of 1000 pg/mL, which was selected as a practical cutoff point, 95% of CRC patients and 88% of FAP patients were successfully identified.

Based on the findings above, we questioned whether the measurement of circulating TXA_2_ could predict the risk of developing CRC. To validate the prognostic value of circulating TXA_2_ levels in CRC, a test study was conducted in both FAP and CRC patients. Results indicated that average circulating TXA_2_ levels in healthy subjects were 284.2 ± 112.0 pg/mL, whereas the average circulating TXA_2_ levels in FAP and CRC patients were 7275.4 ± 4438.6 and 11,328.3 ± 9701.3 pg/mL, respectively (Fig. 2). With a value of 1000 pg/mL selected as a practical cutoff point to discriminate between CRC high-risk and low-risk groups, we successfully identified 21 of 24 FAP patients (88%) and 36 of 38 CRC patients (95%).

### 3.3 Pathophysiological Role of the TXA_2_ Pathway in CRC

**Fig. 3:**
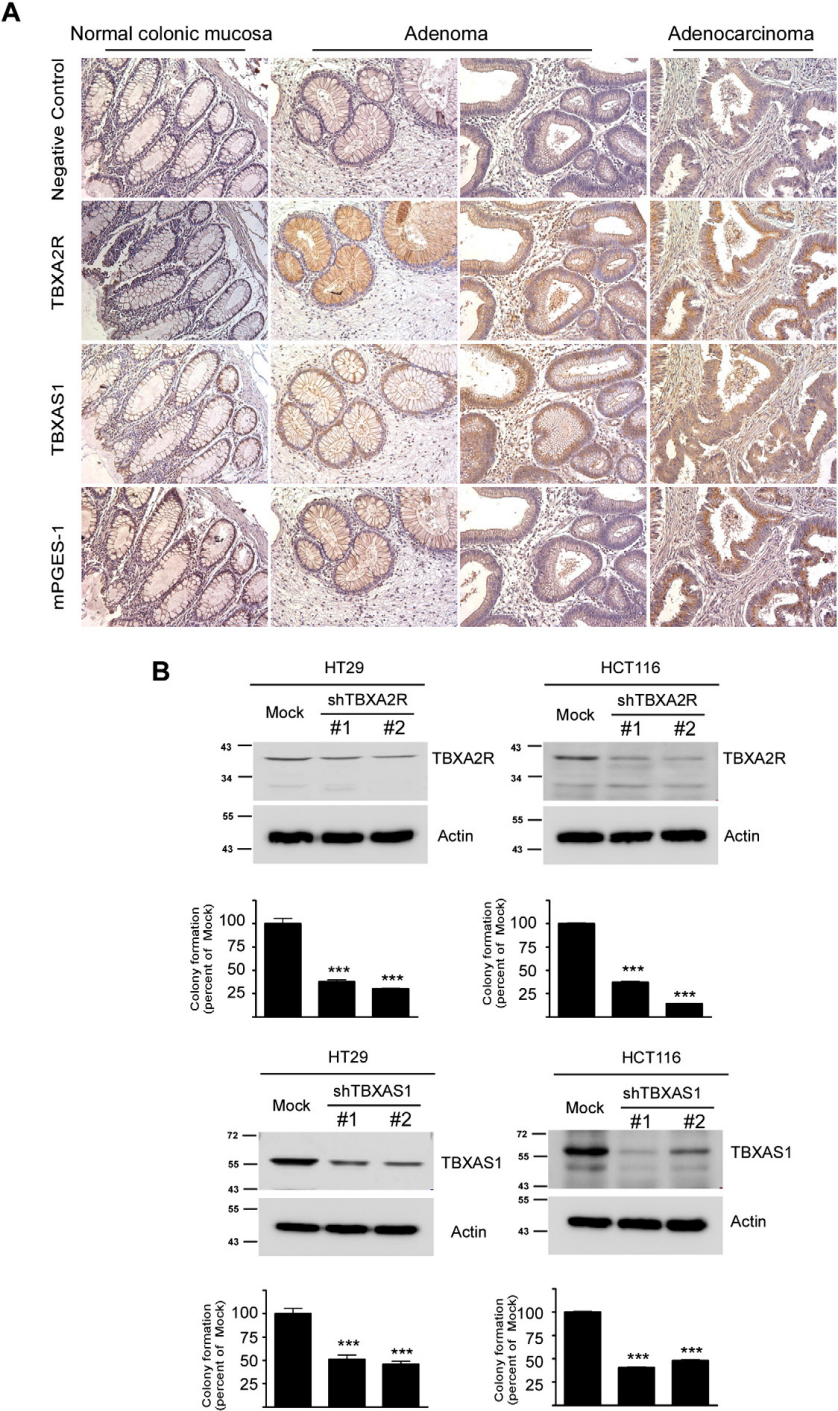
Pathophysiological role of the TXA_2_ pathway in CRC. (A) Immunohistochemical staining of TBXA2R, TBXAS1 or mPGES-1 in biopsy samples, which included normal colonic mucosa, polyps, adenomas, and adenocarcinomas. For antibody-negative controls, the primary antibodies were substituted with normal rabbit serum. Original magnification: 200 ×. (B) The TXA_2_ pathway is associated with tumorigenic properties in human colorectal cancer cells. Knockdown of TBXA2R or TBXAS1 in HT29 or HCT116 colon cancer cells was analyzed by Western blot (upper panels). Mock and knockdown HT29 and HCT116 colon cancer cells were then subjected to anchorage-independent growth assays (lower bar graphs) as described in “Materials and Methods”. The asterisks (***) indicate a significant (*p* < 0.001) decrease in colony formation by knockdown HT29 or HCT116 colon cancer cells.

To clarify the importance of the TXA_2_ pathway in CRC, we examined the expression of the TXA_2_ receptor (TBXA2R) as well as TXA_2_ synthase (TBXAS1, a key enzyme for TXA_2_ biosynthesis) in biopsy samples (Fig. 3A). Our immunohistochemistry staining results clearly showed that both TBXA2R and TBXAS1 were highly expressed in most colonic polyps or tumors, but not in normal colorectal tissues. Importantly, TBXA2R and TBXAS1 were co-localized with each other. Consistent with previous reports regarding the critical role of PGE_2_ in CRC (Castellone et al., 2005, Chulada et al., 2000, Oshima et al., 1996, Sonoshita et al., 2001), we also observed the overexpression of microsomal prostaglandin E synthase-1 (mPGES-1, the rate-limiting enzyme for PGE_2_ biosynthesis) during CRC progression.

Next, we investigated whether the TXA_2_ pathway is directly associated with tumorigenic properties of colon cancer cells. Anchorage-independent growth ability is an ex vivo indicator and a key characteristic of the transformed cell phenotype (Hanahan and Weinberg, 2011). Based on this idea, we confirmed that knockdown of TBXA2R or TBXAS1 in human colorectal cancer cells resulted in fewer colonies being formed in soft agar compared with control cells (Fig. 3B). Collectively, these results suggested that blocking the TXA_2_ pathway might reduce the malignant potential of colon cancer cells.

### 3.4 Aspirin Attenuates CRC in FAP Patients by Targeting the TXA_2_ Pathway

**Fig. 4:**
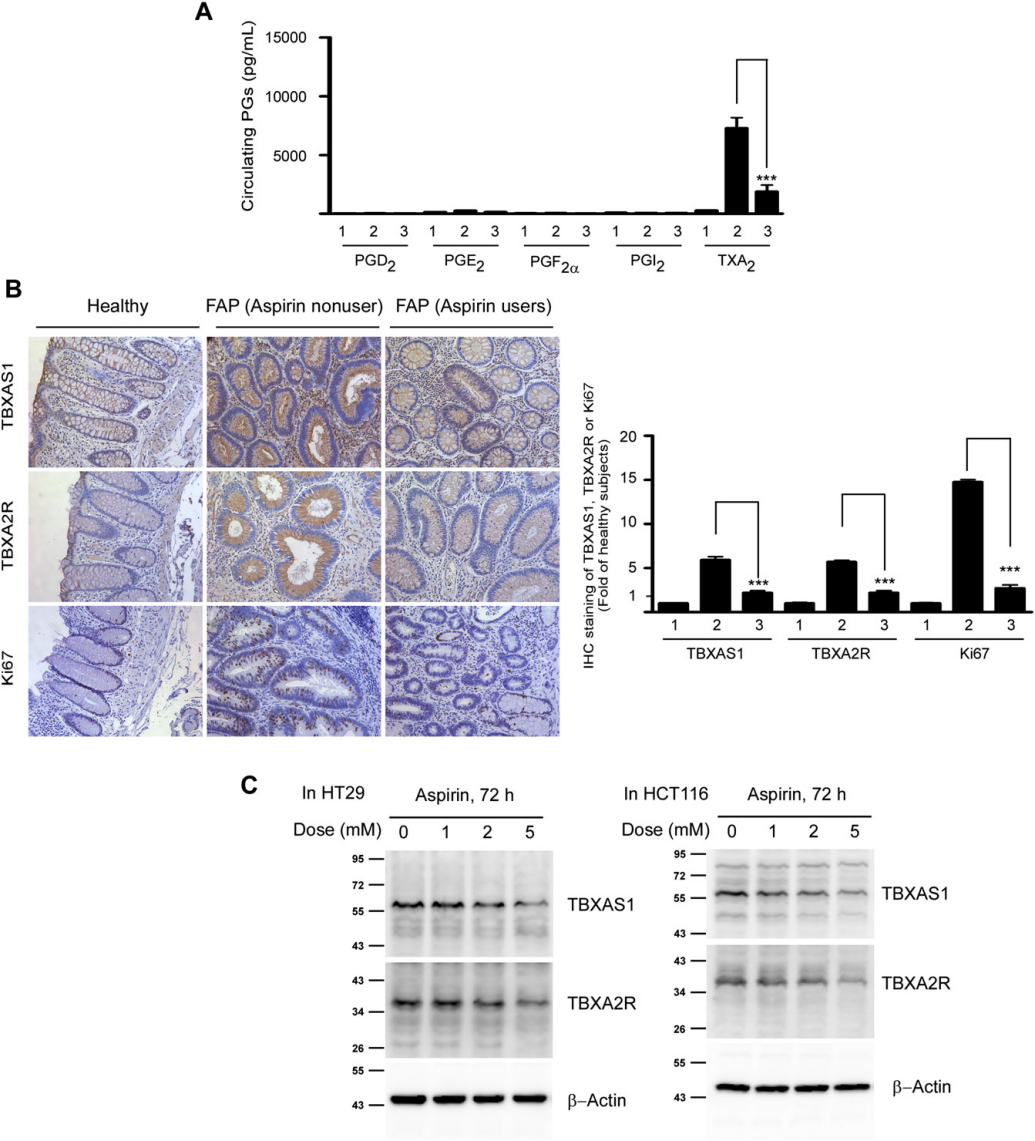
Aspirin reduces CRC risk in FAP patients by targeting the TXA_2_ pathway. (A) Effects of regular aspirin use on circulating PG biosynthesis in FAP patients. 1 = healthy subjects (n = 16); 2 = FAP patients, aspirin nonusers (n = 24); and 3 = FAP patients, aspirin users (n = 14). FAP patients who reported taking two or more standard (325 mg) aspirin tablets per week were classified as regular aspirin users and those taking less aspirin were defined as aspirin nonusers. Data are presented as means ± S.D. The asterisks (***) indicate a significant (*p* < 0.001) decrease in circulating TXA_2_ levels associated with aspirin intake. (B) Effects of regular aspirin use on the expression patterns of TBXA2R, TBXAS1 and Ki-67 in FAP patients. Original magnification: 200 ×. Immunostaining intensities are defined in “Materials and Methods.” (C) Effects of aspirin treatment on TBXA2R and TBXAS1 protein expression in colon cancer cells. HT29 and HCT116 colon cancer cells were treated with aspirin for 72 h. After treatment, cell lysates were subjected to Western blot analysis as described in “Materials and Methods”.

Aspirin shows indisputable promise as a chemopreventive agent against CRC, but its molecular underpinnings remain imperfectly understood (Chan et al., 2007, Algra and Rothwell, 2012, Rothwell et al., 2010). We hypothesized that aspirin might reduce CRC risk by affecting the TXA_2_ pathway. To examine this possibility, we first examined the influence of aspirin intake on circulating PG levels in FAP patients. Results indicated that regular aspirin use significantly decreased the circulating TXA_2_ level in FAP patients, but had little effect on the levels of the other four PGs (Fig. 4A). Due to its very short half-life, TXA_2_ primarily functions in an autocrine or paracrine manner by binding to the TBXA2R, a typical G protein-coupled receptor (GPCR), which might signal platelet aggregation, cell growth and migration (Rothwell et al., 2010). In this study, we found that aspirin intake was associated with lower expression of TBXA2R and TBXAS1, as well as Ki-67, in the epithelial cell from polyps (Fig. 4B). In addition, aspirin treatment down-regulated TBXA2R or TBXAS1 expression in colon cancer cells (Fig. 4C).

## 4 Discussion

In the present study, we provide new evidence showing that the TXA_2_ pathway is involved in CRC pathophysiology. Both TBXAS1 and TBXA2R are highly expressed in colonic neoplastic tissues compared with normal colonic tissues. Knockdown of either TBXAS1 or TBXA2R impairs the anchorage-independent growth capability of colon cancer cells. Importantly, CRC progression is associated with higher circulating TXA_2_ levels, which might merit investigation as a predictor of CRC risk.

Although a large body of evidence indicates that PGE_2_ might be the predominant PG in cancer, the concept that PGE_2_ is the only PG involved in carcinogenesis has long been challenged. For example, PGD_2_ functions as a pro-resolution mediator in ulcerative colitis (Vong et al., 2010), and PGI_2_ is the major PG generated in ovarian epithelial cancer (Daikoku et al., 2005). Here, we provided novel evidence showing that CRC progression is accompanied by a pronounced elevation of systemic TXA_2_ biosynthesis. Notably, in a mouse model of colon cancer, we observed that blood TXA_2_ levels in tumor-bearing mice were 17-fold higher compared to levels in tumor-free mice (Li et al., 2014). Consistent with our findings, Dovizio et al. also reported that FAP patients' urinary 11-dehydro-TXB_2_ (one of the major enzymatic metabolites of TXA_2_) levels were significantly higher compared to those in healthy subjects (Dovizio et al., 2012).

Platelets have long been suspected to be a major source of TXA_2_ in the blood. Based on this idea, we examined platelet counts and found that it was markedly elevated in FAP patients, especially those who had already developed CRC. Importantly, platelet counts were positively correlated with plasma TXA_2_ levels in FAP patients who were aspirin nonusers, but was not associated with those patients who used aspirin regularly (Supplementary Fig. 1). Interestingly, a large body of evidence supports the idea that specific inhibition of COX-1, but not COX-2, greatly affects systemic TXA_2_ biosynthesis (Dovizio et al., 2012, McAdam et al., 1999). Importantly, aspirin exerts its cardio-protective activity by inhibiting platelet-derived COX-1/TXA_2_ biosynthesis. Coincidentally, we confirmed that although overexpressed in FAP patients, COX-2 did not correlate with plasma TXA_2_ levels (Supplementary Fig. 2). Notably, colon cancer cells could trigger platelet activation for TXA_2_ generation (Dovizio et al., 2012). All of these findings, together with our data here, point to the possibility that platelet-derived TXA_2_ plays a functional role in intestinal tumorigenesis. To this end, further studies such as examining susceptibility to intestinal polyps in mice with targeted deletions in either TBXAS1 or TBXA2R are need. Overall, our results indicate that lowering circulating TXA_2_ levels or interfering with the TXA_2_ pathway might be a promising strategy for CRC prevention and/or treatment in the future.

Thrombosis is a common complication in colorectal cancer (CRC) patients, but its molecular mechanisms remain elusive (Sorensen et al., 2000). A dynamic balance between pro-thrombotic TXA_2_ and anti-thrombotic PGI_2_ production is generally accepted to be a contributor to homeostasis of the circulatory system (Cheng et al., 2002). Our data strongly suggested that elevated circulating TXA_2_ levels might be linked with CRC pathophysiology. We also observed that in FAP patients, the levels of circulating TXA_2_ were increased by 25.6-fold compared to healthy subjects, whereas circulating PGI_2_ levels did not change significantly. These findings implied that FAP patients might also be more prone to a risk of cardiovascular disease than healthy subjects. Additional clinical studies are needed to examine this possibility.

Detection of malignant neoplasms at an early stage offers clinical advantages (Srivastava et al., 2001). However, the disturbing reality is that very few reliable biomarkers are available to predict the risk of CRC, one of the most common and deadly cancers. Considering the compliance issues associated with optical colonoscopy and the fecal occult blood test, a large amount of effort is being invested in developing reliable but minimally invasive methods for CRC risk screening. Blood is easily sampled by relatively non-invasive methods, and thus the introduction of a blood-based test could offer an advantage for enhancing patient compliance compared to other tests (deVos et al., 2009). Our findings suggest that circulating TXA_2_ levels might have a potential prognostic or predictive value for the early detection of CRC. Currently, a prospective collective of plasma samples from subjects in a CRC screening guideline-eligible population is underway to further confirm the clinical performance of this biomarker.

Although our findings are promising for translating circulating TXA_2_-based biomarkers from basic research into clinic use, several issues still need to be addressed. For example, our sample size is small, and data collection is only limited in CRC. Thus more rigorous experiments should be conducted to determine biomarker cut-off optimization and calculation of ROC curves. Another issue is how to exclude the possibility of plasma TXA_2_ level confounded by platelet aggregation. To the end, venous blood was carefully collected with anticoagulant in this study. Although, in theory, the possibility that the plasma TXA_2_ levels are confounded by platelet aggregation is very low, measurement of urinary TXA_2_ metabolites such as 11-dehydro TXB_2_ might provide the best estimate of systemic TXA_2_ biosynthesis in vivo. Thus, we confirmed that urinary 11-dehydro TXB_2_ levels were significantly increased in CRC patients compared to healthy subjects (0.83 ± 0.29 versus 5.83 ± 4.22 ng/mg creatinine, respectively, *p* < 0.01) (Supplementary Fig. 3).

In summary, this study established the importance of the TXA_2_ pathway in CRC pathophysiology, and laid the groundwork for introducing TXA_2_-targeted strategy to CRC prevention, early detection and even management.

The following are the supplementary data related to this article.

Supplementary Fig. 1 Platelets are involved in CRC pathophysiology. (A) Platelet count was markedly elevated in FAP patients. Healthy subjects (n = 16); FAP patients without CRC (aspirin nonusers; n = 13); and FAP patients with CRC (aspirin nonusers; n = 12). Data are presented as means ± S.D. The asterisks (***) indicate a significant (*p* < 0.001) increase compared with healthy control subjects. (B) Platelet count and circulating TXA_2_ levels are positively correlated in FAP patients who are aspirin nonusers. Data were analyzed using Prism 5.0 statistical software.

Supplementary Fig. 2 COX-2 expression is not correlated with plasma TXA_2_ levels in FAP patients. (A) COX-2 is overexpressed in FAP patients. Original magnification: 200×. (B) Staining intensity of COX-2 and circulating TXA_2_ levels are not correlated in FAP patients who are aspirin nonusers. FAP patients, aspirin nonusers (n = 13). Data were analyzed using Prism 5.0 statistical software.

Supplementary Fig. 3 Urinary excretion of 11-dehydro TXB_2_ in CRC patients. Urinary 11-dehydro TXB_2_ levels were markedly elevated in CRC patients compared with healthy subjects. Healthy subjects (n = 8); CRC patients (n = 24). Data are presented as means ± S.D. The asterisks (**) indicate a significant (*p* < 0.01) increase compared with healthy control subjects.
