# Supplementary figures and images for: Circulating Prostaglandin Biosynthesis in Colorectal Cancer and Potential Clinical Significance
Source: eBioMedicine. 2014 Dec 9;2(2):165–71. doi: 10.1016/j.ebiom.2014.12.004 (PMC4347518; doi:10.1016/j.ebiom.2014.12.004)

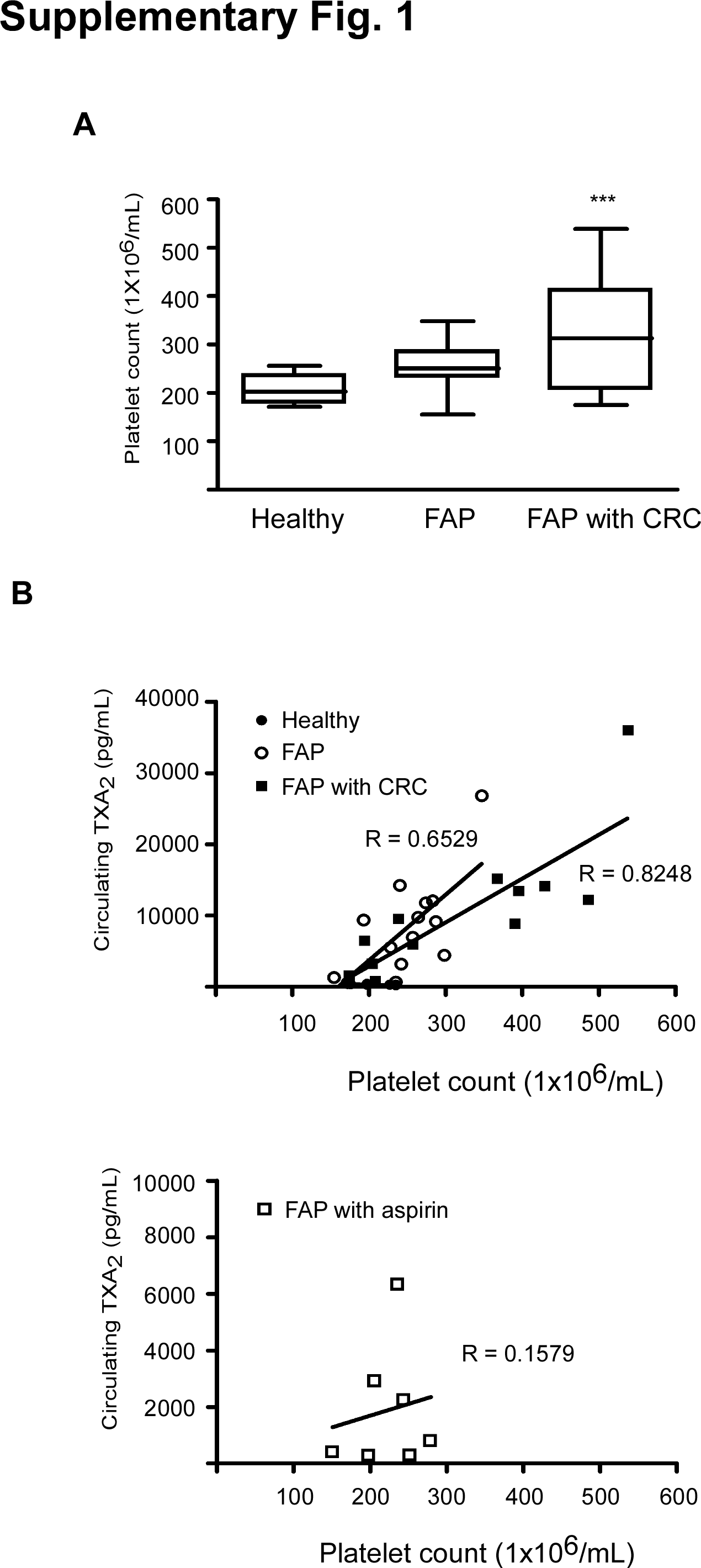

Supplement: Supplementary Fig. 1 — Platelets are involved in CRC pathophysiology. (A) Platelet count was markedly elevated in FAP patients. Healthy subjects (n = 16); FAP patients without CRC (aspirin nonusers; n = 13); and FAP patients with CRC (aspirin nonusers; n = 12). Data are presented as means ± S.D. The asterisks (***) indicate a significant (p < 0.001) increase compared with healthy control subjects. (B) Platelet count and circulating TXA2 levels are positively correlated in FAP patients who are aspirin nonusers. Data were analyzed using Prism 5.0 statistical software. [file mmc1.zip › ebi00044-mmc1.tif]

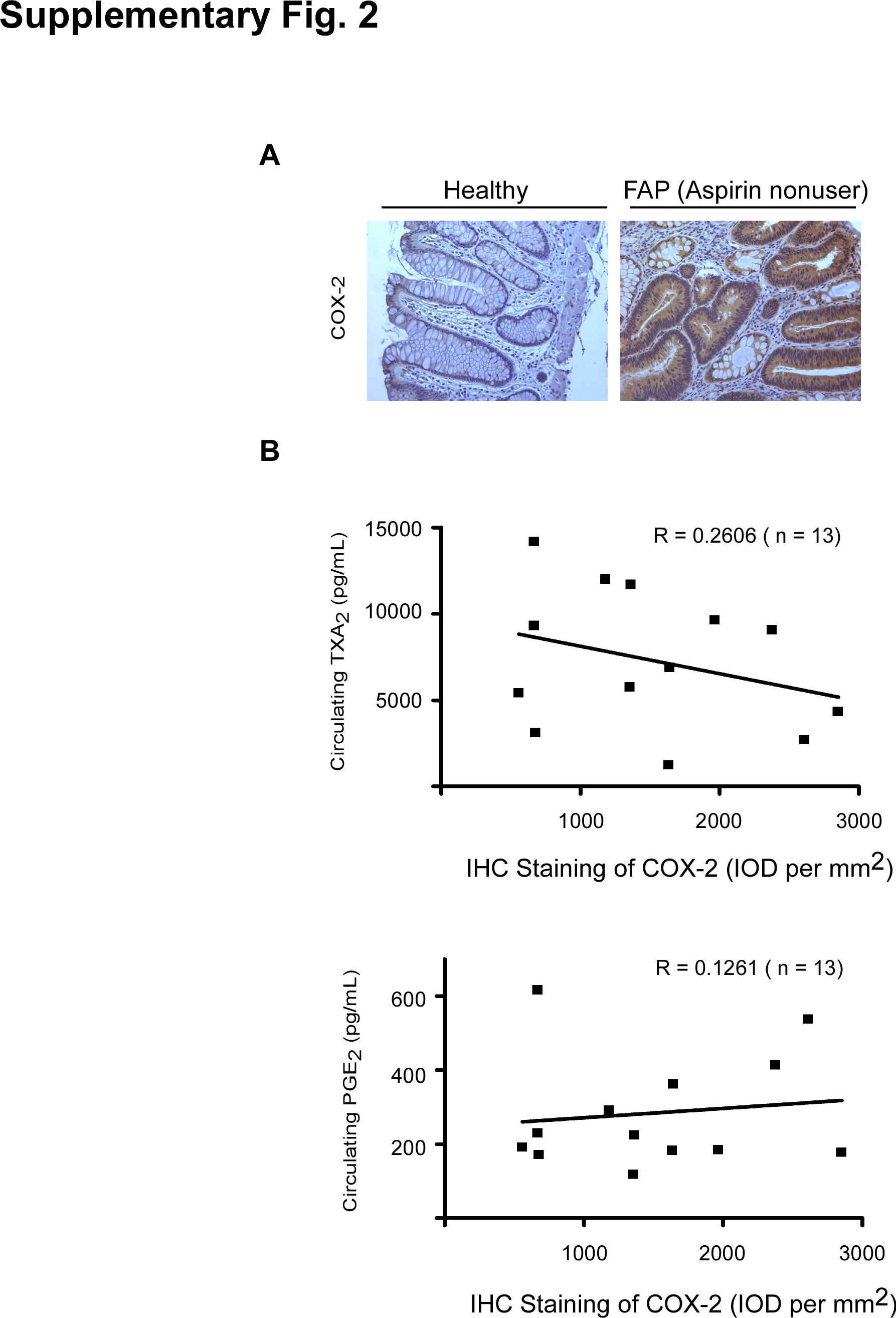

Supplement: Supplementary Fig. 2 — COX-2 expression is not correlated with plasma TXA2 levels in FAP patients. (A) COX-2 is overexpressed in FAP patients. Original magnification: 200×. (B) Staining intensity of COX-2 and circulating TXA2 levels are not correlated in FAP patients who are aspirin nonusers. FAP patients, aspirin nonusers (n = 13). Data were analyzed using Prism 5.0 statistical software. [file mmc2.zip › ebi00044-mmc2.tif]

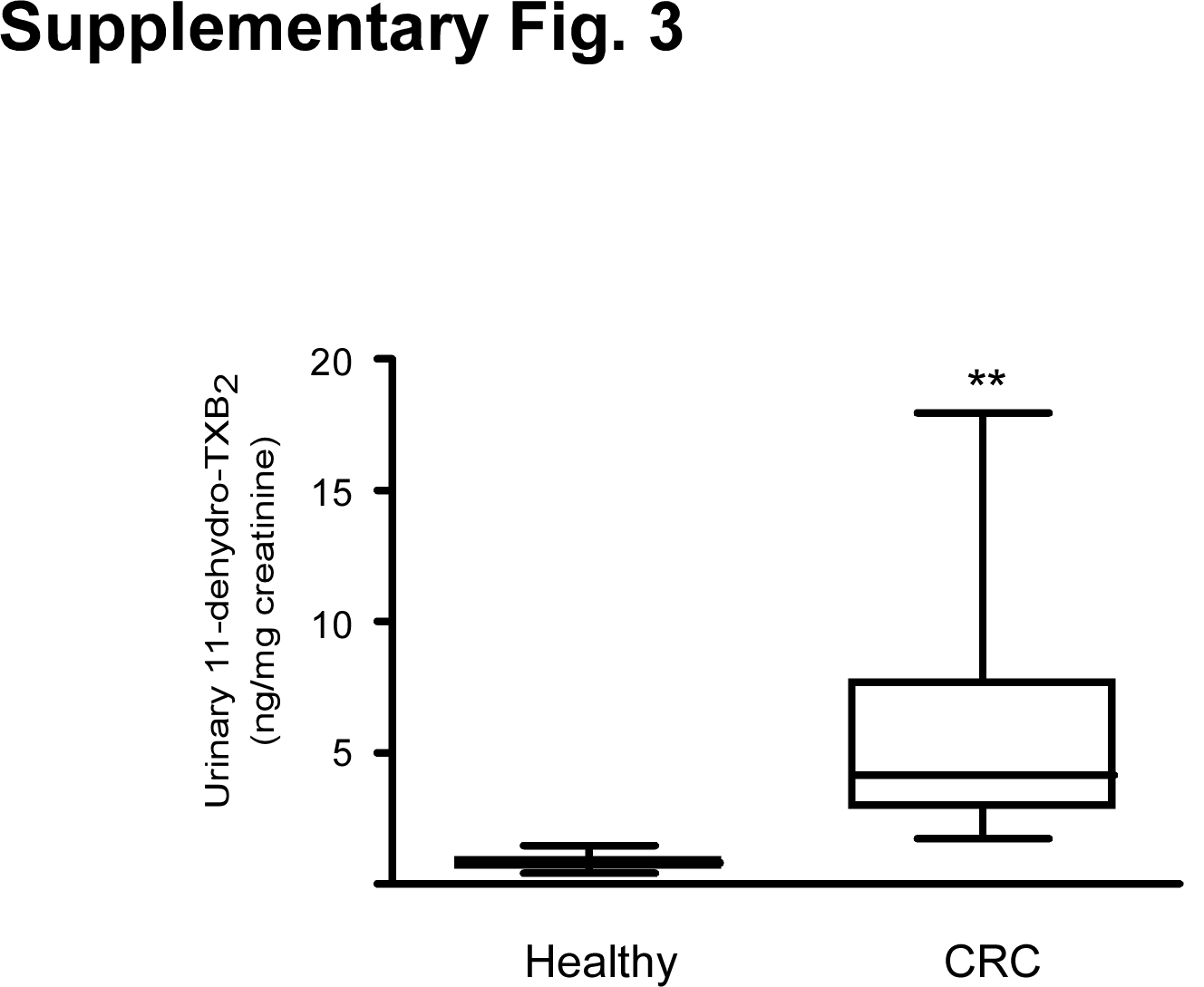

Supplement: Supplementary Fig. 3 — Urinary excretion of 11-dehydro TXB2 in CRC patients. Urinary 11-dehydro TXB2 levels were markedly elevated in CRC patients compared with healthy subjects. Healthy subjects (n = 8); CRC patients (n = 24). Data are presented as means ± S.D. The asterisks (**) indicate a significant (p < 0.01) increase compared with healthy control subjects. [file mmc3.zip › ebi00044-mmc3.tif]
